# Development and Characterization of Indole-Responsive Whole-Cell Biosensor Based on the Inducible Gene Expression System from *Pseudomonas putida* KT2440

**DOI:** 10.3390/ijms23094649

**Published:** 2022-04-22

**Authors:** Paulius Matulis, Ingrida Kutraite, Ernesta Augustiniene, Egle Valanciene, Ilona Jonuskiene, Naglis Malys

**Affiliations:** 1 Bioprocess Research Centre, Faculty of Chemical Technology, Kaunas University of Technology, Radvilėnų pl. 19, LT-50254 Kaunas, Lithuania; paulius.matulis@ktu.lt (P.M.); ingrida.kutraite@ktu.lt (I.K.); ernesta.augustiniene@ktu.lt (E.A.); egle.valanciene@ktu.lt (E.V.); ilona.jonuskiene@ktu.lt (I.J.); 2 Department of Organic Chemistry, Faculty of Chemical Technology, Kaunas University of Technology, Radvilėnų pl. 19, LT-50254 Kaunas, Lithuania

**Keywords:** indole, L-tryptophan, inducible gene expression system, *Pseudomonas putida*, whole-cell biosensor

## Abstract

Indole is a biologically active compound naturally occurring in plants and some bacteria. It is an important specialty chemical that is used as a precursor by the pharmaceutical and chemical industries, as well as in agriculture. Recently, indole has been identified as an important signaling molecule for bacteria in the mammalian gut. The regulation of indole biosynthesis has been studied in several bacterial species. However, this has been limited by the lack of in vivo tools suitable for indole-producing species identification and monitoring. The genetically encoded biosensors have been shown to be useful for real-time quantitative metabolite analysis. This paper describes the identification and characterization of the indole-inducible system *Pp*TrpI/P*_PP_RS00425_* from *Pseudomonas putida* KT2440. Indole whole-cell biosensors based on *Escherichia coli* and *Cupriavidus necator* strains are developed and validated. The specificity and dynamics of biosensors in response to indole and its structurally similar derivatives are investigated. The gene expression system *Pp*TrpI/P*_PP_RS00425_* is shown to be specifically induced up to 639.6-fold by indole, exhibiting a linear response in the concentration range from approximately 0.4 to 5 mM. The results of this study form the basis for the use of whole-cell biosensors in indole metabolism-relevant bacterial species screening and characterization.

## 1. Introduction

Indole is an *N*-heterocyclic aromatic compound that belongs to a large group of naturally occurring biologically active molecules. It plays a signaling role in organisms between various species and kingdoms [1,2]. Importantly, indole has been identified as a significant antimicrobial agent, which can impact the bacterial communication network, decreasing the bacterial resistance, as shown in the example of enteric *Escherichia coli* and *Citrobacter rodentium* infection treatment [3]. Indole-based derivatives also have potential as antibacterial agents against methicillin-resistant *Staphylococcus aureus* [4]. The range of indole applications is wide and versatile, as it can be used either directly or as a precursor to producing pharmaceuticals, amino acid L-tryptophan, plant growth regulators, and dyes [5,6,7,8]. For example, indole-3-butyric acid (3-IBA) is synthesized from indole and is commonly applied as a rooting agent [9]. Notably, biologically active compounds that can be derived from indole, including tryptophan, melatonin, and serotonin, are of high pharmaceutical interest. For instance, indole derivatives are used for the treatment of migraines, depression, various mental disorders, cancer, viral infections, and others [10,11,12,13].

Currently, the main industrial production method of indole and its derivatives is based on Fischer indole synthesis [14]. While the Fischer method is most commonly used, other types of chemical reactions such as Bischler–Möhlau, Hemetsberger–Knittel, Nenitzescu, or Bartoli are used depending on the final desirable product [10,15]. Since the chemical synthesis of indole requires harsh reaction conditions and various by-products generated in the production process are toxic to the environment, alternative bio-based methods are being developed for the synthesis of this compound [4]. *Escherichia coli* and *Vibrio cholerae* possessing tryptophanase (TnaA; EC 4.1.99.1) have been used to produce indole from L-tryptophan [16,17]. However, only up to 5 mM of indole concentrations have been achieved, possibly due to its inhibitory effects on bacteria. Therefore, it is highly beneficial to improve bacterial production by applying metabolic engineering to increase the efficiency of indole biosynthesis and develop microbial strains with improved indole tolerance [4,18]. For instance, the biosynthesis of an indole derivative, the indole-3-pyruvic acid, in *E. coli* has been improved five-fold by introducing and optimizing the expression of *tdiD* (encoding L-tryptophan:phenylpyruvate aminotransferase) from *Aspergillus nidulans* along with other genetic modifications [19]. Furthermore, in the effort to produce bio-indigo from indole, a high-level producing *E. coli* strain has been developed by enhancing the formation of 3-deoxy-D-arabino-heptulosonate 7-phosphate (DAHP) and increasing the gene dosage of *aroG* encoding the DAHP synthase [20]. Advances in the biosynthesis of indole and its derivatives have been reviewed in detail [4].

More than half a century ago, the tryptophan synthase, a final component in the L-tryptophan biosynthesis pathway, was identified [21]. Tryptophan synthases are typically found as heterotetramers (α2β2) in bacteria, plants, and fungi [22]. The α subunit catalyzes the reversible reaction converting indole-3-glycerol phosphate (I3GP) to indole and glyceraldehyde-3-phosphate, whereas the β subunit catalyzes the irreversible condensation of indole and L-serine, forming L-tryptophan. Tryptophan synthase α and β subunits are encoded by *trpA* and *trpB* genes that are part of the *trp* operon in *E. coli* [23]. In *Pseudomonas aeruginosa*, a LysR family transcriptional regulator (TR), encoded by the *trpI* gene, has been shown to activate *trpA* and *trpB* gene expression in the presence of I3GP [24]. Moreover, the *trpA* and *trpB* gene regulation by TrpI has only been identified in *Pseudomonas* family members, such as *P. aeruginosa* and *P. entomophila* [23].

Classical methods that are being applied for the detection of indoles, such as Salkowski, Kovács, and Ehrlich’s reagent assays, or high-performance liquid chromatography (HPLC), possess significant drawbacks. For example, the Salkowski colorimetric assay is non-specific while the HPLC is expensive and time-intensive [25,26,27]. Recently, increasing attention is being directed to the development of whole-cell biosensor-based techniques [28,29] that can be applied as an accurate and relatively inexpensive alternative to the classical methods. In particular, transcription factor-based whole-cell biosensors equipped with fluorescence or luminescence reporters facilitate intracellular and extracellular small molecule quantification in vivo [30,31,32]. Moreover, they enable to perform real-time monitoring of cellular metabolite concentrations [33]. Due to the advantages of these biosensors, it is purposeful to develop a specific whole-cell biosensor and use it for intracellular and extracellular detection of indole.

This paper, for the first time to our knowledge, describes the identification and characterization of a transcription factor-based indole-inducible system from *Pseudomonas putida* KT2440. To facilitate synthetic biology and relevant tool engineering efforts, indole-specific whole-cell biosensors based on *E. coli* and *C. necator* strains are developed. Biosensor response dynamics are parameterized, and their specificity is studied. As this study demonstrates, the indole-inducible system can be applied as a biosensor for screening tryptophanase-positive strains and indole biosynthesis. In principle, it can also be used for studying indole metabolism, screening indole-producing strains, and biotechnology applications.

## 2. Results

### 2.1. Identification of Indole-Inducible System and Biosensor Development

**Figure 1:**
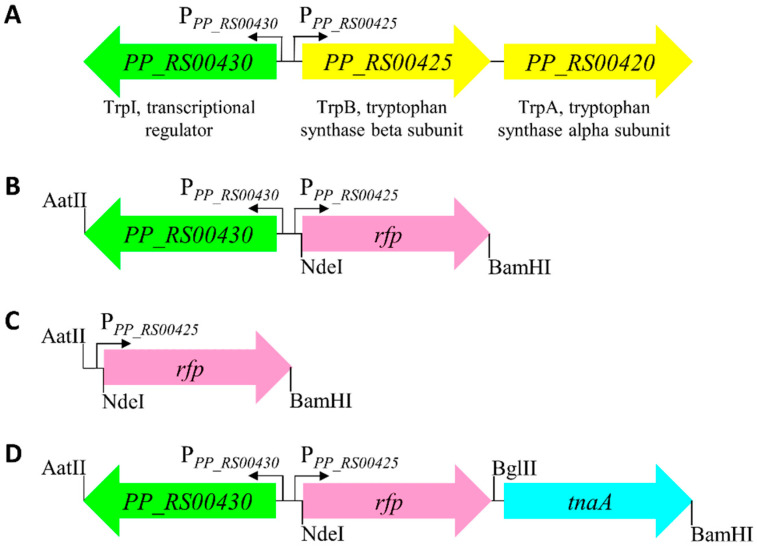
Schematic representation of the gene arrangement of the *trpIAB* operon and its derivatives used in this study. (**A**) The *trpIAB* operon, encoding transcription factor TrpI (locus tag *PP_RS00430*), and tryptophan synthase subunits alpha (TrpA, *PP_RS00420*) and beta (TrpB, *PP_RS00425*) in *P. putida* KT2440. The gene arrangement of (**B**) indole-inducible gene expression system *Pp*TrpI/P*_PP_RS00425_* with *rfp* reporter gene assembled in the construct pPM0081 that was used for developing the whole-cell biosensor, (**C**) intergenic region ‘only’ containing promoter P*_PP_RS00425_* followed by *rfp* gene (pPM0082), and (**D**) inducible system *Pp*TrpI/P*_PP_RS00425_* with *rfp* reporter gene followed by *tnaA* gene encoding tryptophanase (pPM0083). AatII, NdeI, BglII, and BamHI represent positions of restriction sites, which were used for cloning and restriction analysis.

Previously, the transcription factor TrpI has been shown to regulate the expression of tryptophan synthase genes *trpA* and *trpB* in response to the indole derivative I3GP that acts as an inducer in the opportunistic pathogen *P. aeruginosa* [24,34]. In this study, the *trpIAB* operon containing divergently transcribed genes was identified (Figure 1A), and the indole-inducible gene expression system *Pp*TrpI/P*_PP_RS00425_* was proposed in *P. putida* KT2440. This non-pathogenic bacterium, similarly to *P. aeruginosa*, shares the ability to metabolize various aromatic compounds, and, importantly, it is considered a promising strain for industrial applications [35].

**Figure 2:**
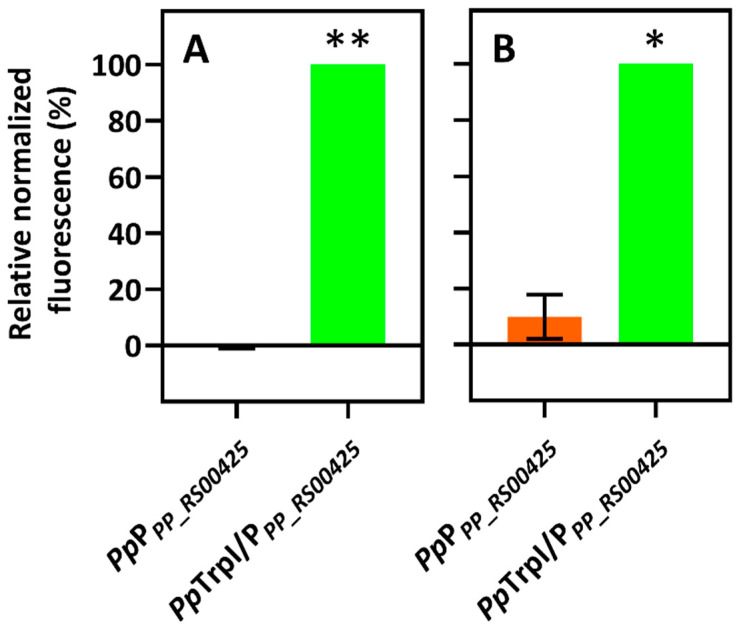
Comparison of biosensors containing *Pp*TrpI/P*_PP_RS00425_* and *Pp*P*_PP_RS00425_* systems. Measurements were carried out using *E. coli-* (**A**) and *C. necator* (**B**)-based biosensors grown in MM and supplemented with 1 mM of indole as an inducer or without inducer. Figure displays relative normalized fluorescence values (in %) at 10 h after supplementation with indole. Data are mean ± SD, n = 3. Asterisks indicate a statistically significant increase of fluorescence in the samples supplemented with inducer comparing to samples without the ligand (** *p* < 0.01, * *p* < 0.02 unpaired two-tailed *t*-test).

Tryptophan synthases are found in most bacteria, and they are present in all known proteobacteria [22], including *E. coli* and *C. necator*. Moreover, the tryptophan synthase α subunit (TrpA) catalyzes reversible conversion between indole and I3GP. Based on this, we reasoned that the putative gene expression system *Pp*TrpI/P*_PP_RS00425_* can be activated in the presence of indole and therefore be used for developing an indole-specific biosensor. To this end, the putative gene expression system *Pp*TrpI/P*_PP_RS00425_*, harboring *trpI* and the adjacent promoter region P*_PP_RS00425_*, was assembled into the vector pBRC1 as described in see Section 4. To demonstrate the wider applicability of the indole-inducible system, the resulting plasmid construct pPM0081 (Figure 1B) was introduced into the gammaproteobacterium *E. coli*, a commonly used bacterial chassis in synthetic biology, and the evolutionary distant betaproteobacterium *C. necator*. The obtained whole-cell biosensors were validated by confirming their response to indole (Figure 2).

Furthermore, to confirm that the transcription factor TrpI regulates the P*_PP_RS00425_* promoter region, the fluorescence outputs of biosensors containing inducible system *Pp*TrpI/P*_PP_RS00425_* (pPM0081) were compared to those of cells harboring construct pPM0082 (Figure 1C) with the *Pp*P*_PP_RS00425_* promoter only. Figure 2 shows that a response to 1 mM indole was observed only in the presence of the *trpI* gene. These results demonstrate that transcription factor TrpI is essential and acts as a transcriptional activator for the inducible gene expression system *Pp*TrpI/P*_PP_RS00425_*. Interestingly, the observed 10% induction using the *C. necator* H16/*Pp*P*_PP_RS00425_* biosensor indicates that some bacteria outside the *Pseudomonas* genus, such as *C. necator*, may possess TR, contributing to the activation of the *Pp*P*_PP_RS00425_* promoter.

### 2.2. Characterization of Indole-Inducible Biosensors

**Figure 3:**
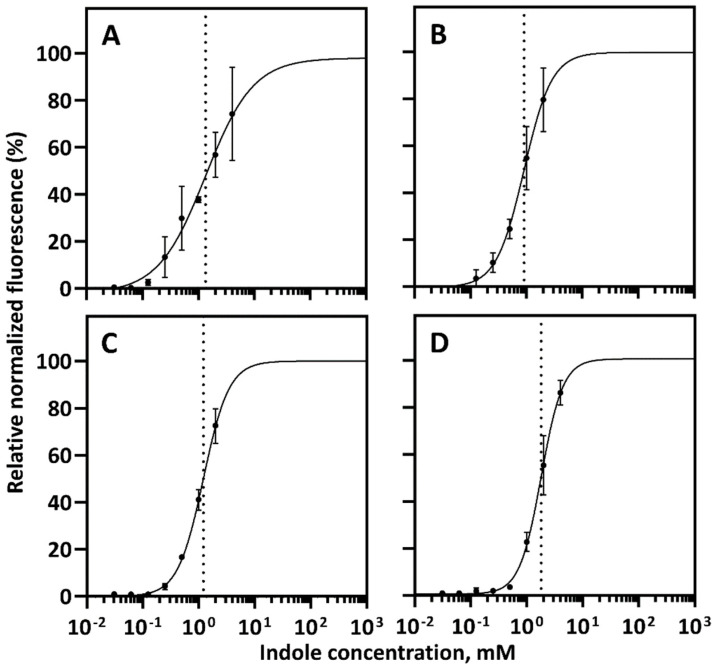
Characterization of inducible system *Pp*TrpI/P*_PP_RS00425_*-based biosensors in response to different concentrations of indole. The *E. coli* (**A**,**C**) and *C. necator* (**B**,**D**) biosensor responses were evaluated in MM (**A**,**B**) or LB medium (**C**,**D**) 4 h after extracellular addition of indole. The dose-responses were fitted using the Hill function (for more details see Section 4). The maximum reporter output *b*_max_ was set to 100%. The inducer concentration that mediates half-maximal reporter output *K*_m_ is indicated by a dotted line. Data are mean ± SD, n = 3.

The indole-inducible gene expression system *Pp*TrpI/P*_PP_RS00425_* (pPM0081) was subsequently characterized by evaluating its response to different concentrations of indole (dose-response) and dynamic range of induction as described previously [36,37]. The fluorescence and absorbance outputs were measured over time using both *E. coli* and *C. necator*-based biosensors containing the inducible system *Pp*TrpI/P*_PP_RS00425_* in Luria-Bertani (LB) medium and minimal medium (MM) supplemented with different concentrations of indole. For both biosensors, the inducible system’s activation, represented by increased fluorescence output compared to the uninduced state, starts 60 min after the addition of indole (Supplementary Figure S1). Notably, the induction kinetics profiles differ between *E. coli* and *C. necator*-based biosensors, most likely due to variability in their growth rates and responses to the indole. *E. coli* growth was less affected by the indole, with no significant growth inhibition up to 1 mM, which is a commonly occurring concentration of this compound in the intestinal tract [4]. However, *C. necator* was more susceptible to the indole, with significant growth inhibition observed at concentrations of 0.125 mM and above. Figure 3 shows the dose-response curves representing the correlation between extracellular indole concentration and relative normalized fluorescence 4 h after inducer supplementation. Data indicate that biosensors can be tuned in the range of approximately 0.4 to 5 mM for a linear fluorescence output.

*E. coli*- and *C. necator*-based biosensors exhibited a dynamic range of 639.6- and 11.9-fold in MM or 373.5- and 101.4-fold in a rich LB medium, respectively (Table 1). Notably, the observed variability of the dynamic range between *E. coli* and *C. necator* biosensors in a rich and minimal medium can be attributed to the indole’s signaling and antimicrobial properties, which may have a differential effect on the cell growth and/or indole transport of diverse bacterial species. A lower dynamic range of *E. coli/Pp*TrpI*/*P*_PP_RS00425_* in a rich medium than that in MM can be caused by traces of indole or structurally similar compounds that might be present in LB and interact with the transcription factor TrpI. Whereas the significantly lower dynamic range of *C. necator/Pp*TrpI*/*P*_PP_RS00425_* in LB and MM than that of *E. coli/Pp*TrpI*/*P*_PP_RS00425_* is likely associated with the indole degradation observed in several bacterial species [4]. Indeed, a decrease in fluorescence output was observed at the later stage of the time course experiment (between 8 and 12 h after the addition of indole) compared to the *E. coli* biosensor (Supplementary Figure S1A,B), indicating consumption of indole by *C. necator*. A similar response attributable to the inducer’s consumption has been previously reported for acrylate-inducible systems [38]. Moreover, a protein sequence similarity search using BLAST (www.ncbi.nlm.nih.gov; accessed on 1 March 2022) showed that *C. necator* H16 contains the gene cluster *H16_RS17385-H16_RS17400* encoding enzyme homologues responsible for indole conversion into anthranilate as reported for *Acinetobacter* sp. O153 and *Cupriavidus* sp. SHE [39,40]. The low dynamic range of *C. necator/Pp*TrpI*/*P*_PP_RS00425_* in MM could also be associated with the catabolic repression proposed previously for aromatic compounds [37]. Notwithstanding the above, similar indole concentrations are required to mediate the gene expression from inducible system *Pp*TrpI*/*P*_PP_RS00425_* in both types of biosensors and media with *K*_m_ values ranging from 0.9 to 1.8 mM.

### 2.3. Specificity of Indole-Inducible Biosensors

**Figure 4:**
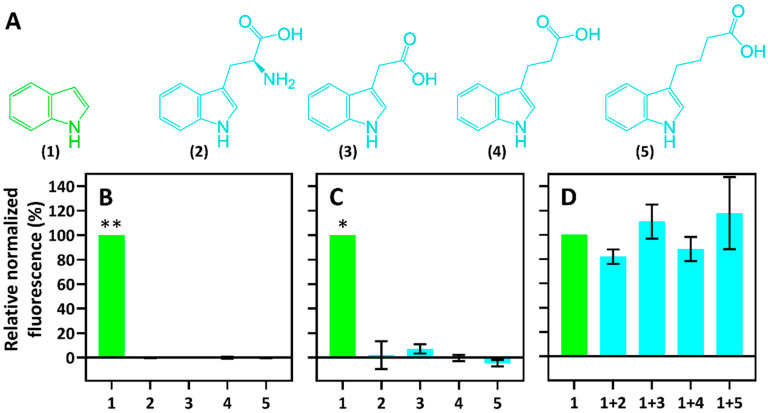
Specificity of *Pp*TrpI/P*_PP_RS00425_*-based whole-cell biosensors. (**A**) Compounds that were tested for induction of the gene expression system *Pp*TrpI/P*_PP_RS00425_*: indole (1), L-tryptophan (2), 3-IAA (3), 3-IPA (4), 3-IBA (5). Percentage values of relative normalized fluorescence after 10 h of addition of each compound at 1 mM are displayed for whole-cell biosensors based on either *E. coli* (**B**) or *C. necator* (**C**). (**D**) An analysis of *E. coli*/*Pp*TrpI/P*_PP_RS00425_* biosensor’s repression by structurally similar compounds with 1 + 2, 1 + 3, 1 + 4, and 1 + 5 representing equimolar mixtures of indole and structurally similar compounds (each at a concentration of 1 mM). Measurements were carried out in MM. Data are mean ± SD, n = 3. Asterisks indicate a statistically significant increase in fluorescence in the samples supplemented with inducer comparing to samples without the ligand (** *p* < 0.01, * *p <* 0.015, unpaired two-tailed *t*-test).

To evaluate the specificity, the inducible system *Pp*TrpI/P*_PP_RS00425_* -based whole-cell biosensors were tested for induction with compounds that are structurally similar to indole. These included the indole metabolism product L-tryptophan (2) and phytohormones such as indole-3-acetic acid (3-IAA) (3), indole-3-propionic acid (3-IPA) (4), and indole-3-butyric acid (3-IBA) (5) (Figure 4A). RFP fluorescence assays using these compounds were carried out. To this end, *E. coli*- and *C. necator*-based biosensors harboring the inducible system *Pp*TrpI/P*_PP_RS00425_* (pPM0081) were cultivated in MM. The fluorescence and absorbance outputs were monitored 10 h after supplementation with each compound to a final concentration of 1 mM, and relative normalized fluorescence values were calculated as % of the normalized fluorescence obtained by using 1 mM indole. Results revealed that neither L-tryptophan (2), 3-IAA (3), 3-IPA (4), nor 3-IBA (5) induces the system *Pp*TrpI/P*_PP_RS00425_* with a confidence level of more than 99.0 and 98.5% for *E. coli*- and *C. necator*-based biosensors, respectively (Figure 4B,C). Therefore, it can be concluded that the developed biosensors exhibit a strong affinity for their primary ligand indole and are strictly specific.

Furthermore, to evaluate if L-tryptophan (2), 3-IAA (3), 3-IPA (4), or 3-IBA (5) can competitively repress indole-mediated activation of the inducible system *Pp*TrpI/P*_PP_RS00425_*, the *E. coli*-based biosensor was subjected to the ligand competition assays in MM (Figure 4D). Fluorescence measurements using equimolar mixtures of ligands showed that none of the structurally similar compounds tested had a significant effect on the biosensor’s response to indole, suggesting that they do not directly interfere with the binding of indole to the transcription factor TrpI and induction of *Pp*TrpI/P*_PP_RS00425_*.

### 2.4. Application of Whole-Cell Biosensor

Indole is produced from the aromatic amino acid L-tryptophan by tryptophanase (TnaA) [41]. Deletion of the *tnaA* gene has previously been shown to improve L-tryptophan biosynthesis in *E. coli* [42]. A protein sequence similarity search using BLAST revealed no homologue in *C. necator* H16. Besides, its genome contains the gene cluster *H16_RS17385-H16_RS17400* encoding enzyme homologues responsible for indole consumption, as reported previously for *Acinetobacter* sp. O153 and *Cupriavidus* sp. SHE [39,40]. Therefore, we reasoned that the heterologous expression of the *tnaA* gene in this bacterium could allow us to monitor the transient indole synthesis and its accumulation in the cell.

**Figure 5:**
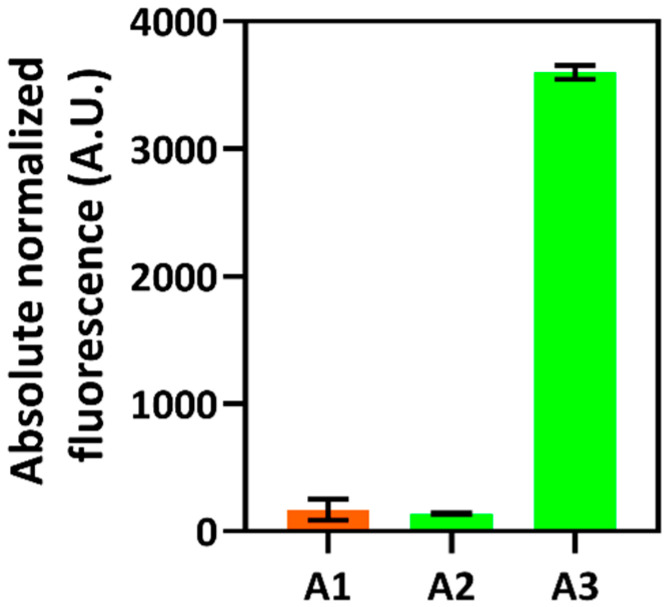
Screening of *tnaA*-positive strains. Absolute normalized fluorescence of *C. necator* strains harboring inducible system *Pp*TrpI/P*_PP_RS00425_* only (control, A1) and inducible system *Pp*TrpI/P*_PP_RS00425_* with *tnaA* gene (A2 and A3). Data are mean ± SD, n = 3.

The biosensor developed on the basis of *C. necator* was applied to screen bacterial strains possessing the functional *tnaA* gene. Cells were transformed with the plasmid pPM0083 (Figure 1D). This construct contained the *tnaA* gene from *E. coli* (locus tag *b3708*), which was cloned downstream and transcriptionally coupled to the inducible gene expression system *Pp*TrpI/P*_PP_RS00425_*, enabling simultaneous synthesis and detection of indole. Subsequently, *C. necator* H16 cells harboring the plasmid pPM0083 were inoculated into the MM and were grown overnight. The resulting cultures were then subjected to the measurement of absorbance and fluorescence (Supplementary Figure S2). Figure 5 shows elevated fluorescence levels for strain A3, indicating the biosynthesis and accumulation of indole intracellularly and, therefore, the presence of functional gene *tnaA*, whereas low fluorescence levels in the case of strain A2 show either the absence of tryptophanase activity or rapid consumption of indole in this strain background. Further research will be required to identify the genetic differences between strains A2 and A3 and if A2 accumulated spontaneous mutations, resulting in an indole-negative phenotype.

Overall, these results show that the developed biosensor is applicable for the screening of microbial strains possessing tryptophanase activity. Moreover, it can potentially assist with monitoring the expression of indole metabolism-relevant genes, screening enzyme variants, and evaluating of their catalytic properties.

## 3. Discussion

Indole has attracted much attention due to its widespread presence in the natural environment and various wastes, including byproducts of petroleum, tobacco, farming, and mining industries [43]. Importantly, it is widely used in pharmaceuticals, polymers, dyes, agro, and other chemicals. TR-based whole-cell-bacterial biosensors have found application in environmental microbiology, synthetic biology, and biotechnology [30,31,32,33]. However, very little research has been performed on developing such tools for indole detection and monitoring. In this study, we developed and characterized biosensors that respond to indole in a concentration-dependent manner. To achieve this, we identified the inducible system *Pp*TrpI/P*_PP_RS00425_* in *Pseudomonas putida* KT2440. The construct containing the TR gene (*trpI*), promoter region, and *rfp* reporter gene was successfully assembled and introduced into *E. coli* and *C. necator* bacterial cells, resulting in whole-cell biosensors based on two different classes of bacteria, gammaproteobacteria and betaproteobacteria, respectively. Using these biosensors, we confirmed that TrpI is essential for the regulation of the P*_PP_RS00425_*-dependent inducible gene expression system. It needs to be mentioned that the TrpI protein belongs to the LysR-type family of prokaryotic transcriptional regulators [17] and has previously been shown to bind I3GP, resulting in gene expression activation [18].

Furthermore, developed indole-inducible biosensors were thoroughly characterized by assessing their dynamic range and dose-dependence. A very high dynamic range of 639.6-fold was observed using an *E. coli*-based biosensor in MM. Despite the moderate level of sensitivity with *K*_m_ values in the mM concentration range, both biosensors showed a high degree of specificity to indole when compared to L-tryptophan (2), 3-IAA (3), 3-IPA (4), and 3-IBA (5). The indole biosensor’s utility was further exemplified by its application for tryptophanase-positive strain screening.

Recently, Herud-Sikimić and colleagues have shown that a genetically encoded biosensor for 3-IAA enables real-time monitoring of the uptake and disposal of auxin by individual cells and within cell sections in planta [44]. However, with few reports focused on indole derivatives, no indole-specific whole-cell biosensor has been described in the literature to date [44,45]. This study shows that our developed biosensor is specific to indole and can be used to measure the intracellular and extracellular concentrations of this compound. Future work can be focused on the application of this tool for the monitoring of indole concentration in the environment, industrial wastewater, or sewage [46,47]. In addition, it has the potential to be used for (a) screening enzymes and microbial strains exhibiting different L-tryptophan and indole specific catalytic properties, (b) evaluating flux in indole producing or degrading bacteria, (c) measuring indole transport and cross-species communication, and (d) indole metabolism-relevant metabolic engineering and biotechnology applications.

## 4. Materials and Methods

### 4.1. Chemicals

High purity chemicals used as ligands for assaying whole-cell biosensors were purchased from Sigma-Aldrich and included the following: indole (cataloge no. I3408), indole-3-acetic acid (I2886), indole-3-propionic acid (I5386), indole-3-butyric acid (220027), and L-tryptophan (T0254).

### 4.2. Bacterial Strains and Media

All strains employed in this study are listed in Supplementary Table S1. For cloning and plasmid propagation, *E. coli* Top10 (Thermo Fisher Scientific, Waltham, MA, USA) was used. To characterize indole-inducible systems and determine dose-response, dynamic range, and specificity of biosensors, RFP fluorescence assays were performed using *E. coli* Top10 and *C. necator* H16 strains. Bacterial cells were grown in either Luria-Bertani (LB) medium or minimal medium (MM). MM for *C. necator* included 1 g/L NH_4_Cl, 9 g/L Na_2_HPO_4_·12H_2_O, 1.5 g/L KH_2_PO_4_, 0.2 g/L MgSO_4_·7H_2_O, 0.02 g/L CaCl_2_·2H_2_O and 0.0012 g/L (NH_4_)_5_[Fe(C_6_H_4_O_7_)_2_] with 1 mL/L trace element solution SL7 (1.3 mL/L 25% (*w*/*v*) HCl, 0.07 g/L ZnCl_2_, 0.1 g/L MnCl_2_·4H_2_O, 0.062 g/L H_3_BO_3_, 0.190 g/L CoCl_2_·6H_2_O, 0.017 g/L CuCl_2_·2H_2_O, 0.024 g/L NiCl_2_·6H_2_O and 0.036 g/L Na_2_MoO_4_·2H_2_O) supplemented with 0.4% (*w*/*v*) sodium gluconate. *E. coli* was grown in M9 MM containing 200 mL/L of microelement solution M9 (64 g/L Na_2_PO_4_·7H_2_O, 15 g/L KH_2_PO_4_, 2.5 g NaCl, 5 g NH_4_Cl), 2 mM MgSO_4_, 0.1 mM CaCl_2_, 1 µg/mL thiamine hydrochloride, 0.4 mM L-leucine and 0.4 % glucose. For recovery after transformation of *C. necator*, SOC medium (Sigma-Aldrich, St. Louis, MO, USA) supplemented with 20 mM glucose was used. Chloramphenicol was added to the medium when required at the following concentrations: 25 µg/mL or 50 µg/mL for *E. coli* and *C. necator*, respectively. Solid medium was prepared by supplementation with 15 g/L agar.

### 4.3. Cloning and Transformation

Purification of plasmid DNA was performed by employing the GeneJET Plasmid Miniprep Kit (Thermo Fisher Scientific, Waltham, MA, USA). Microbial genomic DNA was obtained by exploiting the GenElute Bacterial Genomic DNA Extraction Kit (Sigma-Aldrich, St. Louis, MO, USA). For cloning, DNA was amplified by PCR in 20 µL reactions using the Phusion High-Fidelity DNA polymerase (Thermo Fisher Scientific Baltics, Vilnius, Lithuania). To obtain the gel-purified linearized DNA, Zymoclean Gel DNA Recovery Kit was used. The NEBuilder HiFi DNA Assembly Master Mix was purchased from New England Biolabs, Ipswich, MA, USA. Restriction enzymes, T4 DNA Ligase, and DreamTaq DNA polymerase were purchased from Thermo Fisher Scientific Baltics, Vilnius, Lithuania. The reactions for PCR, digestion, and ligation were set up as per the manufacturer’s protocols.

With some modifications to standard protocol, *E. coli* transformations were performed by mixing 50 µL of formerly prepared chemically competent *E. coli* Top10 with 50 ng plasmid DNA, incubating on ice for 5 min, carrying out the heat shock at 42 °C for 1.5 min and subsequently incubating on ice for 5 min [48]. The recovery of transformed cells was achieved by incubation in 1 mL of LB medium (Thermo Fisher Scientific, Waltham, MA, USA) at 37 °C for 1 h. The transformants were plated on LB agar containing the respective antibiotic and incubated overnight at 37 °C.

Transformations of *C. necator* were carried out by adding 100 ng plasmid DNA to 100 µL of electrocompetent *C. necator* H16 in a pre-chilled electroporation cuvette (0.2 cm gap width, Bio-Rad). The mixture was incubated on ice for 5 min [49], following the electroporation at 2.5 kV using a Micropulser (Bio-Rad, Hercules, CA, USA). The recovery of the transformed cells was performed by incubation in 1 mL of SOC medium at 30 °C for 2 h. After recovery, the transformants were plated on LB agar containing the respective antibiotic and incubated at 30 °C for 2 days.

### 4.4. Plasmid Construction

Oligonucleotide primers were synthesized by Thermo Fisher Scientific (Supplementary Table S2). Plasmids were constructed by employing the NEBuilder HiFi DNA Assembly method following manufacturer’s protocol (New England Biolabs, Ipswich, MA, USA). All plasmid constructs were verified by Sanger sequencing (Eurofins Genomics, Ebersberg, Germany), PCR amplification, or restriction analysis using endonucleases AatII, BamHi, BglII, and NdeI [44].

Indole-inducible system *Pp*TrpI/P*_PP_RS00425_* was PCR amplified using oligonucleotide primers P015 and P016 from *P. putida* KT2440 genomic DNA. The PCR product containing *trpI* gene and *PP_RS00430*/*PP_RS00425* intergenic region (Supplementary Figure S3A) was cloned into AatII- and NdeI-linearized vector pBRC1 that was constructed as pEH006 in [50]. Resulting plasmid was designated as pPM0081 (Figure 1B). The *PP_RS00430*/*PP_RS00425* intergenic region containing indole-inducible promoter *Pp*P*_PP_RS00425_* only (Supplementary Figure S3B) was PCR amplified with oligonucleotides P015TgSr and P016 from *P. putida* KT2440 genomic DNA and cloned as above into pBRC1, resulting in plasmid pPM0082 (Figure 1C).

Plasmid pPM0083 was assembled to contain both indole-inducible system *Pp*TrpI/P*_PP_RS00425_* and *tnaA* gene encoding the tryptophanase (Figure 1D and Supplementary Figure S3C). The *tnaA* was PCR amplified using oligonucleotide primers P065 and P066 from *E. coli* MG1655 genomic DNA. PCR fragment was inserted into pPM0081 using BamHI restriction site and placing *tnaA* in direct orientation downstream to the *rfp* gene.

### 4.5. Fluorescence and Absorbance Measurements

For determination of normalized fluorescence, the RFP fluorescence and cell culture absorbance were measured at multiple time points. In assay preparation, freshly grown bacterial cells were used to inoculate 2 mL of LB medium or MM containing appropriate antibiotics in 50-mL conical centrifuge tubes, and cell cultures were grown overnight at 200 rpm and 30 °C. To obtain logarithmically growing cells, *E. coli* and *C. necator* overnight cultures were diluted to 0.05 and 0.1 OD_600_, respectively, in 5 mL of fresh medium with antibiotic. After the cells were grown to an OD_600_ of 0.15–0.2, culture aliquots of 142.5 µL were transferred into 96-well plate (flat and clear bottom, black; Corning Incorporated, Corning, NY, USA) and 7.5 µL of indole was added to each mini culture to the required final concentration. Absorbance and fluorescence of mini cultures were measured using an Infinite^®^ M Nano+ microplate reader (Tecan, Grödig, Austria). Multiple time point measurements were taken every 10 min over the course of 12 h with a plate reader measuring RFP fluorescence at excitation and emission wavelengths set to 585 nm and 620 nm, respectively, and absorbance at 600 nm. The fluorescence gain factor was assigned to 120%. Obtained fluorescence and absorbance values were corrected by subtracting the autofluorescence and absorbance of the culture medium. The absolute normalized fluorescence was determined dividing the fluorescence value by corresponding absorbance value.

The dose-response of biosensors was determined by using single time-point values of fluorescence and absorbance, which were obtained from the multiple time-point measurements 4 h after supplementation of indole at different concentrations ranging from 0.02 to 2.5 mM. Similarly, the fluorescence and absorbance data for biosensor specificity analysis or tryptophanase-positive strain screening were obtained from the multiple time-point measurements as a single time-point 10 h after addition of ligand at 1 mM or unknown concentration, respectively. For all assays, the cultures of *E. coli* and *C. necator* were prepared as described above, and 142.5 µL of logarithmically growing cells, supplemented with 7.5 µL of ligand, were cultured in 96-well plate. The fluorescence and absorbance were measured using the same conditions and plate reader as indicated above.

### 4.6. Non-Linear Least-Squares Fitting and Dynamic Range Calculation

To perform a non-linear least-squares fit using the Hill function, the absolute normalized fluorescence values were analyzed and plotted as a function of ligand concentration using software GraphPad Prism 9 as follows:

(1) $$\mathrm{RFP}\left(I\right)=b_{max}\times \frac{I^{h}}{K_{m}^{h}+I^{h}}+b_{min}$$

where RFP(*I*)—absolute normalized fluorescence value at given ligand concentration *I*; *b_max_* and *b_min_—*the maximum and minimum levels of reporter output in absolute normalized fluorescence units, respectively; *h—*the Hill coefficient; *K_m_—*the inducer concentration, corresponding to the half-maximal reporter’s output.

The dynamic range *μ* was calculated using Formula (2) as follows:

(2) $$\mu =\frac{b_{max}}{b_{min}}$$

## Figures and Tables

**Table 1:** Parameters of biosensors based on the indole-inducible system *Pp*TrpI/P*_PP_RS00425_*.

Whole-Cell Biosensor	Growth Conditions	Dynamic Range, fold	*K*_m_, mM
*E. coli/Pp*TrpI*/*P*_PP_RS00425_*	LB medium, 30 °C	373.5	1.207
*E. coli/Pp*TrpI*/*P*_PP_RS00425_*	Minimal medium, 30 °C	639.6	1.347
*C. necator/Pp*TrpI*/*P*_PP_RS00425_*	LB medium, 30 °C	101.4	1.819
*C. necator/Pp*TrpI*/*P*_PP_RS00425_*	Minimal medium, 30 °C	11.9	0.9055

Data are mean of three biological replicas. *K*_m_ represents the indole concentration required to achieve the half-maximal activation of inducible system.

## Supplementary Materials

The supplementary materials are available online at https://www.mdpi.com/article/10.3390/ijms23094649/s1.
