# Supplementary material for: Development and Characterization of Indole-Responsive Whole-Cell Biosensor Based on the Inducible Gene Expression System from Pseudomonas putida KT2440
Source: Int J Mol Sci. 2022 Apr 22;23(9):4649. doi: 10.3390/ijms23094649 (PMC9105386; doi:10.3390/ijms23094649)
Supplement: Supplementary file 1 [file ijms-23-04649-s001.zip › ijms-1684185-supplementary.pdf]

Supplementary Materials

Development and characterization of indole-responsive whole-cell biosensor based on the inducible gene expression system from *Pseudomonas putida* KT2440

Paulius Matulis [1], Ingrida Kutraite [1], Ernesta Augustiniene [1], Egle Valanciene [1], Ilona Jonuskiene [1] and Naglis Malys [1,2,*]

[1] Bioprocess Research Centre, Faculty of Chemical Technology, Kaunas University of Technology, Radvilėnų pl. 19, Kaunas LT-50254, Lithuania

[2] Department of Organic Chemistry, Faculty of Chemical Technology, Kaunas University of Technology, Radvilėnų pl. 19, Kaunas LT-50254, Lithuania

*Corresponding author, email: naglis.malys@ktu.lt

Supplementary Tables

Supplementary Table S1. Bacterial strains used in the study.

Strain	Characteristics	Reference or source
Escherichia coli TOP10	F^- *mcrA* Δ(*mrr-hsdRMS-mcrBC*) Φ80*lacZ*ΔM15 Δ*lacX*74 *recA*1 *araD*139 Δ(*araleu*)7697 *galU* *galK* *rpsL* (StrR) *endA1* *nupG*	Thermo Fisher Scientific, Waltham, MA, USA
Cupriavidus necator H16	Wild-type strain	DSM 428 (DSMZ, Braunschweig, Germany)

Supplementary Table S2. Oligonucleotide primers used in the study.

Name	Oligonucleotide primer sequence (5′ → 3′)
P015	gggcctttcgttttatgacgtctgatatgcaagcgaaactaagcgctctgc
P016	cgtcttcgctactcgccatatgggcgctccaaggcgtag
P015TgSr	gggcctttcgttttatgacgtcttacctgtgagtttttctgacaagtttac
P065	tcactccaccggtgcttaagcaaaagatctttaagaaggagatatacatatggaaaactttaaacatctccctgaaccg
P066	tccttactcgagtttggatccttaaacttctttaagttttgcggtgaagtgac

Restriction endonuclease recognition sites are underlined.

Supplementary Figures

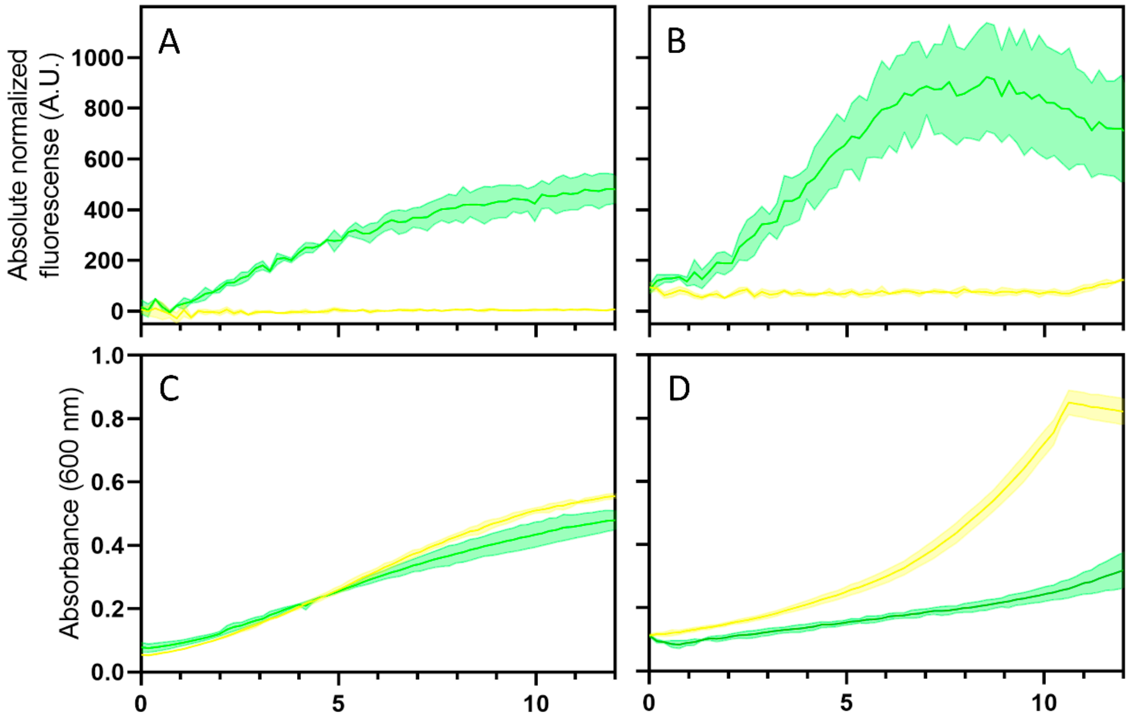

Supplementary Figure S1. Induction dynamics of *Pp*TrpI/P$_{PP_RS00425}$-based *E. coli* and *C. necator* biosensors. Absolute normalized fluorescence (A, B) and absorbance (C, D) obtained using *E. coli* (A, C) and *C. necator* (B, D) harbouring inducible system *Pp*TrpI/P$_{PP_RS00425}$ (pPM0081). Measurements were performed over the period of 12 hours in MM supplemented with 1 mM (green) and without indole (yellow). Data are mean ± SD, *n* = 3.

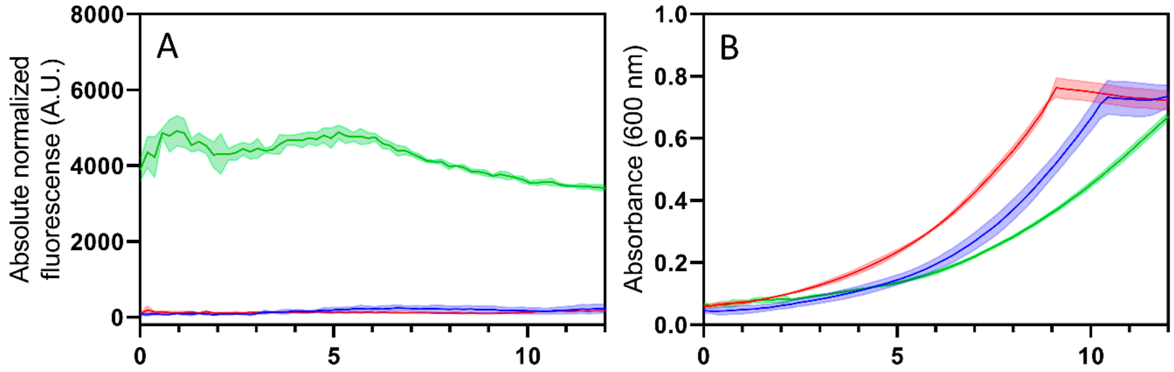

Supplementary Figure S2. Screening and characterization of *tnaA*-positive strains using *C. necator*/*Pp*TrpI/P$_{PP_RS00425}$ biosensor. Absolute normalized fluorescence (A) and absorbance (B) of *C. necator* harbouring inducible system *Pp*TrpI/P$_{PP_RS00425}$ without (pPM0081; control, blue) or with *tnaA* gene (pPM0083; red and green). Data are mean ±SD, n=3.

A

AatII

gacgtctgatatgcaagcgaaa**cta**agcgctctgccgcgcaagttccgcccttaaccactgcgccaactgctcggcgcgcccgtctgcggcgcgccgtggcacccacaatgc

end of *trpI* (*PP_RS00425*)

cagcgccgcctgggtaggtgaaaagccccaaggcgcactcaatcggcccgcccgcaggtcatcggcgaccagcggctgcggtgctatcgccacgcccaatccggccacg
gcagcctccagcaggtaatacaggtgctcgaaggcctggccatagtgaatggccgccggctccaggccctgttgcaccacccaggtcggccaggcttgtgggcgtgacgt
ggtgtgcagcaaggcttcgcccagcagtgccttggcgggcgcgccctgcaagcgctggaaacctgcgaaatgcgggctgagcaccggcccgatgcattcctcggccagca
catgcacctgcatgtccgctggccacggtggctcggcatacaccagcaacgcatccagcccgggccgccgcgggtcaaggtcgccctcacccgccgacaggtgcaagcgc
agctcaggcaaatccgcctgcaaccgccccagccgcggaataaaccaccgcgccagcaggctgcccgagcagcccagcacgaatggcgcctcgctcacatcacggctga
gctcggaacacacgctgcgcagccggtcgaaggcctcaccgctggcatcgcgcagacgcataccggcatctgtgagtttgatgccacgcccgtccttgacgaacagggcta
cgcccagatgctcctcaagcaccttgatctgccggctgacagcgccgtgggtgacgtgcagcgcttccgccgcctggctgacactgttcagcctggctgtagcctcgaaggc
gcgcagggcattgaggggagggaggtcgtgggc**cat**tttacctgtgagtttttctgacaagtttacgcaatcttatcggttttcagccgagcttgccgtggttagagtaaagcc

start of *trpI*

NdeI

catcactcattcactacgccttggagcgcccat**atg**

start of *rfp*

B

AatII

gacgtctttacctgtgagtttttctgacaagtttacgcaatcttatcggttttcagccgagcttgccgtggttagagtaaagcccatcactcattcactacgccttggagcgcc

NdeI

cat**atg**

start of *rfp*

C

BglII

agatctttaagaaggagatatacat**atg**gaaaactttaaacatctccctgaaccgttccgcattcgtgttattgagccagtaaaacgtaccactcgcgcttatcgtgaagaggc

start of *tnaA*

aattattaaatccggtatgaacccgttcctgctggatagcgaagatgtttttatcgatttactgaccgacagcggcaccggggcggtgacgcagagcatgcaggctgcgatga
tgcgcggcgacgaagcctacagcggcagtcgtagctactatgcgttagccgagtcagtgaaaaatatctttggttatcaatacaccattccgactcaccagggccgtggcgca
gagcaaatctatattccggtactgattaaaaaacgcgagcaggaaaaaggcctggatcgcagcaaaatggtggcgttctctaactatttctttgataccacgcagggccatag
ccagatcaacggctgtaccgtgcgtaacgtctatatcaaagaagccttcgatacgggcgtgcgttacgactttaaaggcaactttgaccttgagggattagaacgcggtattg
aagaagttggtccgaataacgtgccgtatatcgttgcaaccatcaccagtaactctgcaggtggtcagccggtttcactggcaaacttaaaagcgatgtacagcatcgcgaag
aaatacgatattccggtggtaatggactccgcgcgctttgctgaaaacgcctatttcatcaagcagcgtgaagcagaatacaaagactggaccatcgagcagatcacccgcg
aaacctacaaatatgccgatatgctggcgatgtccgccaagaaagatgcgatggtgccgatgggcggcctgctgtgcatgaaagacgacagcttctttgatgtgtacaccga
gtgcagaaccctttgcgtggtgcaggaaggcttcccgacatatggcggcctggaaggcggcgcgatggagcgtctggcggtaggtctgtatgacggcatgaatctcgact
ggctggcttatcgtatcgcgcaggtacagtatctggtcgatggtctggaagagattggcgttgtctgccagcaggcgggcggtcacgcggcattcgttgatgccggtaaact
gttgccgcatatcccggcagaccagttcccggcacaggcgctggcctgcgagctgtataaagtcgccggtatccgtgcggtagaaattggctctttcctgttaggccgcgatc
cgaaaaccggtaaacaactgccatgcccggctgaactgctgcgtttaaccattccgcgcgcaacatatactcaaacacatatggacttcattattgaagcctttaaacatgtgaa

BamHI

agagaacgcggcgaatattaaaggattaacctttacgtacgaaccgaaagtattgcgtcacttcaccgcaaaacttaaagaagtt**taa**ggatcc

end of *tnaA*

Supplementary Figure S3. Nucleotide sequences of genetic elements of the indole-inducible system. (A) Nucleotide sequence containing *trpI* gene, encoding LysR family transcriptional regulator (locus tag *PP_RS00430*), and intergenic region *PP_RS00430*/*PP_RS00425*, assembled in pPM0081 and corresponding to the schematic representation in Figure 1B. (B) Sequence of intergenic region *PP_RS00430*/*PP_RS00425* (in pPM0082 and Figure 1C). (C) Nucleotide sequence of *tnaA* gene encoding tryptophanase (in pPM0083 and Figure 1D). The start and end of open reading frames are displayed in bold. AatII, NdeI, BglII, and BamHI restriction sites are underlined. The nucleotides of intergenic region *PP_RS00430*/*PP_RS00425* are highlighted in green.
